# Gene selection tool (GST): A R-based tool for genetic disorders based on the sliding-window proportion test using whole-exome sequencing data

**DOI:** 10.1371/journal.pone.0185514

**Published:** 2017-09-28

**Authors:** Sugi Lee, Minah Jung, Jaeeun Jung, Kunhyang Park, Jea-Woon Ryu, Jeongkil Kim, Dae-soo Kim

**Affiliations:** 1 Department of Bioinformatics, KRIBB School of Bioscience, Korea University of Science and Technology(UST), Daejeon, Korea; 2 Department of Rare Disease Research Center, Korea Research Institute of Bioscience & Biotechnology(KRIBB), Daejeon, Korea; 3 Core Facility Management Center, Korea Research Institute of Bioscience & Biotechnology(KRIBB), Daejeon, Korea; German Cancer Research Center (DKFZ), GERMANY

## Abstract

Whole-exome sequencing (WES) can identify causative mutations in hereditary diseases. However, WES data might have a large candidate variant list, including false positives. Moreover, in families, it is more difficult to select disease-associated variants because many variants are shared among members. To reduce false positives and extract accurate candidates, we used a multilocus variant instead of a single-locus variant (SNV). We set up a specific window to analyze the multilocus variant and devised a sliding-window approach to observe all variants. We developed the gene selection tool (GST) based on proportion tests for linkage analysis using WES data. This tool is R program coded and has high sensitivity. We tested our code to find the gene for hereditary spastic paraplegia using SNVs from a specific family and identified the gene known to cause the disease in a significant gene list. The list identified other genes that might be associated with the disease.

## Introduction

With the development of next-generation sequencing (NGS), a large amount of genetic information can be obtained at a low cost. The progress of high-throughput sequencing has enabled whole-exome sequencing (WES) to directly identify candidate variants, particularly in the case of limited family members. However, for WES, the list of candidate variants can be quite large, including false positives caused by sequencing errors. The genome-wide association study method, which compares data with reference data, is predominantly applied; however, such data for family-specific diseases remain unavailable [1].

Classically, linkage analysis has been used to analyze family-specific diseases. Using the concept of crossover and recombination, the allele combination transmitted to the offspring can be identified, and the likelihood ratio test statistic, LOD, which uses siblings’ and parents’ genomic information, can be calculated [2–4]. For more detailed analysis, various models such as maximum LOD (MLOD), maximum non-parametric linkage score (MNPL), maximum heterogeneity LOD (MHLOD), maximum Kong and Cox linear extension (MKC(lin)) of NPL, and maximum Kong and Cox exponential extension (MKC(exp)) of NPL based on LOD have been developed [5,6]. Generally, families share similar genomic data; therefore, it is difficult to find a specific gene of interest because many candidates are selected. It is more difficult to find the gene for a particular disease affecting fewer family members [5,7,8].

In linkage analysis, researchers look for genes that are passed on to the next generation without crossover, and they are interested in the recombination fraction between two loci. To this end, data from siblings or parents are used. It is difficult to calculate the recombination fraction when complete family information is not available.

The SNVs of small families are generally analyzed by the multilocus linkage test, which is more powerful than the single-locus [9]. Therefore, we propose a method to compare the SNVs of each family member at a specific locus and quantify it, and to increase the SNV information of a small family using the sliding-window method to determine whether the family has a specific disease. The SNV pattern for each position was digitized to simplify the calculation, and it was converted to R code, enabling fast calculation of large SNV data. Therefore, this approach enables quick analysis even in a low-end environment.

### System and method

Using the SNV pattern, we aimed at finding genes causing rare diseases in certain families. We grabbed the window range to see the effect of the SNVs, and slid it by one position to see its effect continuously. We devise the gene selection tool (GST) in R based on this concept. The GST procedure is combined in two steps, as follows:

### Step 1: Calculation of the SNV pattern frequency

Let *S* be the disease state of a family member. *S* = 0 is set as a normal person, *S* = 1 patient, and the *j—th* SNV of the *v—th* family member is set as *SNV*_*jv*_(*S*). *REF*_*j*_ is the *j*–*th* reference, where *j* = 1,2,…,*J*, and *v* = 1,2,…,*V*. *J* is total number of SNVs, and *V* is the total number of family members. To calculate the SNV pattern frequency, family data might be handled according to two conditions.

**Condition 1. All SNV information of both parents is available**. If parent SNV information is available, we set the father with *v* = 1 and mother with *v* = 2. An indicator function is classified according to the disease state of an offspring as follows:

$$I\left(SNV_{jv}\left(0\right)\right)=\{\begin{array}{l} 0,\;|\;REF_{j}=SNV_{jv=1,2}\left(0\right)=SNV_{jv\neq 1,2}\left(0\right)\neq SNV_{jv=1,2}\left(1\right)\\ 1,\;\;\;\;|Otherwise \end{array}$$

$$I\left(SNV_{jv}\left(1\right)\right)=\{\begin{array}{l} 0,\;|\;REF_{j}=SNV_{jv=1,2}\left(0\right)\neq SNV_{jv\neq 1,2}\left(1\right)=SNV_{jv=1,2}\left(1\right)\\ 1,\;\;\;\;|Otherwise \end{array}$$

*Where v = 3*,…,*V*.

Genotype is considered when calculating the SNV pattern. In this same disease state, the value of the indicated function is zero, and we are interested in this position.

Let *LF*_*j*._ be the whole family’s *j—th* SNV pattern frequency. Then, the equation is as follows: *LF*_*j*._ = *∑*_*v = 3*,…,*V*_ *I*(*SNV*_*jv*_), *0≤ LF*_*j*._ *≤ C = V*-2, where *C* is the number of offspring.

**Condition 2. SNV information of only one parent is available, or no SNV information is available for either parent**. Under condition 2, all family members are regarded as offspring. Of course, it is desirable to distinguish between the presence and absence of SNV information of a parent. In the end, however, the result of the calculation is the same. This is because the conditions of SNV pattern frequency are the same, so there is no difference between the two results in step 2. The SNV pattern should be calculated on the assumption that the patient differs from the reference, and a normal person is the same as the reference. Based on this, we can derive an indicator function of a normal person and patient as follows:

$$I\left(SNV_{jv}\left(0\right)\right)=\{\begin{array}{l} 0,\;|\;REF_{j}=SNV_{jv}\left(0\right)\\ 1,\;\;\;\;|Otherwise \end{array}$$

$$I\left(SNV_{jv}\left(1\right)\right)=\{\begin{array}{lll} 0, & | & REF_{j}\neq MSNV_{j}\left(S=1\right)=SNV_{jv}\left(1\right)\\ 0.5, & | & REF_{j}\neq MSNV_{j}\left(S=1\right)\neq SNV_{jv}\left(1\right)\\ 1, & \;\;| & Otherwise \end{array}$$

*Where MSNV*_*j*_(*S* = 1) is the most common pattern in a patient’s SNV at the *j—th* position and *v = 1*,…,*eV645V*.

Finally, the whole family’s *j—th* SNV pattern frequency *LF*_*j*._ is as follows: *LF*_*j*._ = *∑*_*v = 1*,…,*V*_ *I*(*SNV*_*jv*_), 0*≤ LF*_*j*._ *≤*.*V*,…,*V ollow* is the number of offspring. When patients and normal people are well distinguished in the pattern of the *j—th* SNVs, *LF*_*j*._ would be close to zero. Therefore, GST use the window method to ascertain where the *LF*_*j*._ pattern frequency is close to zero. This is explained in more detail in step 2.

### Step 2: Calculation of the score

The window with *n* SNV markers before and after the *i—th* SNV is designated as ℶ(*i*,*n*) = *i*-*n*, *i*-*n*+1,…,*i*-1,*i*, *i*+1,…,*i+n*-1,*i+n*, *where i = n*+1,*n*+2,…,*U*-*n*-1. The *v—th* family member’s *j—th* central SNV pattern proportion in the window is defined as *PR*_*jv*_ = *∑*_*j*∈ℶ(*i*,*n*)_*I*(*SNV*_*jv*_)*/*(*2n*+1), and the probability of the whole family’s *j—th* central SNV pattern proportion is defined as *PR*_*j*._ = *∑*_*j*∈ℶ(*i*,*n*)_*LF*_*j*._*/C*(*2n*+1), *j = n*+1,*n*+2,…,*U-n*-2,*U-n*-1. To find the significant SNV, we decided to use a one-tailed proportion test of *PR*_*j*._ under the null hypothesis *PR*_*j*._≥0.5 at a significance level *α* = 0.05 for each chromosome. Here, GST have to find a gene that leads to the rejection of the null hypothesis. Because of the similarity of the SNV pattern in the same disease state, the *PR*_*j*._ approaches zero. To see these patterns in succession, GST slid from the window at the (*n*+1)*–th* SNV to the (*U-N*-1)*–th* SNV. The locus of SNVs within the window also plays an important role. Despite sharing similar pattern frequencies with other windows, if the SNVs in a window are positioned closer to each other, the results obtained will be more distinguished. The weight system was selected to account for such variations in distribution of the loci. Hence, we chose the weight system. Let *w*_*j*_(*n*,*t*) = {*L*(*SNV*_*max*(*j*∈ℶ(*i*,*n*))._)*-L*(*SNV*_*min*(*j*∈ℶ(*i*,*n*))._)}*/t*, where *L*(*SNV*_*j*._) is the locus at *SNV*_*j*._ and *t* is the tuning parameter measured in 1 mega base pairs (Mb). Let *f*(*PR*_*j*._) and *w*_*j*_ (*n*,*t*) be the p-values of *PR*_*j*._ and weight value in the window, respectively. Finally, the *j—th* SNV’s score is

$$Score_{j}=-log\left(w_{j}\left(n,t\right)\cdot f\left(PR_{j}\right)\right),$$

*where j* ∈ ℶ(*i*, *n*).

At the significance level *α* = 0.05, GST selected SNVs with *Score*_*j*_ greater than—log(0.05) = 2.996 and *j—th* SNV pattern frequency *LF*_*j*._ = 0. Selected SNVs have a 95% probability of being a variant. Among them, the SNV present at the gene position located in the coding DNA sequence (CDS) or splice site was finally selected. Finally, the gene name at the locus of the selected SNV was provided with the value of the score, description and phenotype information. In addition, scores of SIFT and PolyPhen-2 are presented together as a result. Here, the results are placed above the gene with the phenotype information, and the gene with no information is placed down. GST program coded have been provided in the S1 File (GST Code).

## Window size

Generally, there is no fixed method for determining the window size in the sliding-window method. Since the method we propose is based on a one-tailed proportion test, we determined the window size by applying the method of determining the sample size in the proportion test. Assuming that the *LF*_*j*._ in the window is a sample, it can be substituted into the equation that determines the existing sample size, as follows:

$$n=\left(\left({\left(z_{0.05}\cdot 0.5\right)}^{2}/\left(C\cdot E^{2}\right)\right)-1\right)/2$$

where *z*_0.05_ = 1.645 is the critical value of the normal distribution, and *E* = 0.05 is the margin of error. The above equation can be used to determine the appropriate window size without simulation. However, the window size *n* is constant. If the window size *n* is not a constant, it is rounded off to a constant value. It is included in the R code and is automatically calculated according to the number of families.

## Implementation

We analyzed the actual data to see whether the GST can identify genes affecting a rare disease. We used a family tree of two sisters with hereditary spastic paraplegia, a rare disease. The disease indicates progressive spasticity and contraction in the lower limbs. Details of the family have been provided in the S1 Fig. In this family, clinical trials have already been completed, and the *SPAST* gene has been revealed as a significant causative gene. We executed the GST with these data to determine whether *SPAST* was selected as a statistically significant candidate gene with the methods proposed by us. The window size *n* was decided as 19 according to the formula for determining the window size. The above data analysis took about 15 min when run as R code. It requires R version 3.3.3 and has been successfully tested on Windows 7 with 24 GB RAM.

**Fig 1:**
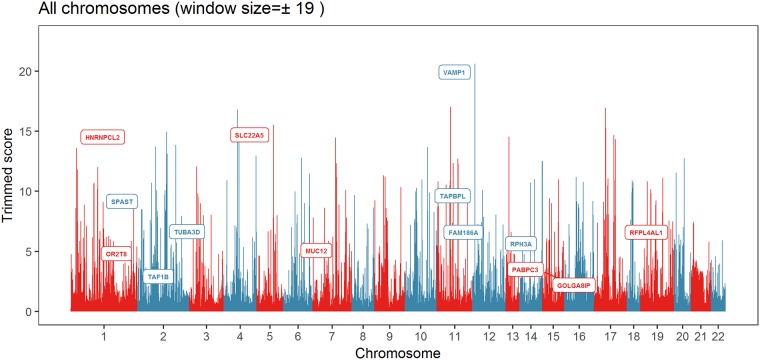
A plot of the significant gene list of all chromosomes except X and Y chromosomes. To distinguish the chromosomes, they are expressed in red and blue. The candidate genes of Table 1 are represented by gene names and their trimmed score values. Details of the trimmed score option have been provided in the S1 File (GST User guide).

Table 1 and Fig 1 shows the results of the analysis, with 14 significant candidate genes. The overall result of the description, phenotype, and score of SIFT and PolyPhen-2 appear in the S2 File (GST result). Among them, *SPAST*, which was found through clinical experiments, was selected. The *SPAST* gene is a member of the ATPases associated with a variety of cellular activities (AAA) protein family, which share an ATPase domain. They are involved in a variety of cellular processes including membrane trafficking, intracellular motility, organelle biogenesis, protein folding, and proteolysis. Mutation associated with *SPAST* is considered to be the cause of autosomal dominant paroxysmal paralysis 4. In addition, the *VAMP1* gene was selected as well, which is also known to be a gene responsible for hereditary spastic ataxia disease, similar to hereditary spastic paraplegia. We compared the result of GST with the Variant Annotation, Analysis & Search Tool (VAAST). Only one *SPAST* gene was selected when using the VAAST. Both methods found *SPAST* gene. The detailed result of VAAST is in the S2 File (VAAST result). The difference in the presence of the remaining genes is due to algorithmic differences. VAAST uses an algorithm based on 1000 genome database. GST is only an algorithm to find family-specific genes. Here, the high score of GST has two meanings. First, because of family data, there may be a family-specific gene that appears similar to another gene. Second, it may be an important disease-related gene. Both scenarios have important implications for a disease-affected family. Therefore, it would be desirable to conduct further studies based on the list of genes provided by the GST.

**Table 1:** A list of significant genes deduced by GST analysis.

Rank	Chromosome	Locus	Gene	Score
1	chr12	6575044	*VAMP1*	20.610176
2	chr5	131705949	*SLC22A5*	15.517678
3	chr1	13183015	*HNRNPCL2*	13.602401
4	chr12	6562285	*TAPBPL*	10.389608
5	chr2	32341282	*SPAST*	8.452735
6	chr19	56283297	*RFPL4AL1*	7.497583
7	chr2	132236963	*TUBA3D*	5.867672
8	chr7	100638484	*MUC12*	5.788482
9	chr12	50749294	*FAM186A*	5.750429
10	chr12	113319600	*RPH3A*	4.951603
11	chr1	248084909	*OR2T8*	4.101806
12	chr13	25671700	*PABPC3*	4.087220
13	chr2	10059844	*TAF1B*	3.596389
14	chr15	23261850	*GOLGA8IP*	3.281563

## Conclusions

Linkage analysis is re-emerging as an extremely useful method in WES data analysis, particularly for the identification of causal rare variants in inherited disorders. Identification of candidate variants requires sifting through a large number of variants, including false positives generated by sequencing errors. Accordingly, we proposed a systematic approach to select causative genes through linkage analysis of variants extracted from WES data. In the case of rare diseases, the amount of information is particularly insufficient in the case of small families. We quantified the multilocus SNV pattern in the window to facilitate the calculation, and the amount of information was found to be sufficient. Moreover, we applied the sliding-window algorithm to analyze all possible SNVs and observe the distribution of results from each locus. Ultimately, the final score was obtained by weighting the results obtained from the sliding window using the SNV locus. If the score is higher than the defined boundary and the SNV pattern of the family is divided into patients and controls, and concurrently, the central SNV’s position of the window is located in the CDS or splice site, the result is presented as a significant candidate. We confirmed that our method is reliable and time efficient by using actual clinical data of rare diseases. We expect that the GST will be able to analyze SNVs of small families having rare diseases. In the future, we plan to further improve the performance of our GST to more accurately reduce the list of candidate genes that affect certain rare diseases.

## Supporting information

S1 Fig A pedigree of simulation data. The person whose back-ground color is expressed in black is the patient, and the person expressed in white is the normal person. The person represented by the dotted line has no data.
(TIF)

S1 File R file to implement the GST. It is a R file that contains R code, sample data and user guide.
(ZIP)

S2 File Result file to implement the GST and VAAST. It is a excel file that contains results of GST and VAAST.
(ZIP)
