# Supplementary figures and images for: Gene selection tool (GST): A R-based tool for genetic disorders based on the sliding-window proportion test using whole-exome sequencing data
Source: PLoS One. 2017 Sep 28;12(9):e0185514. doi: 10.1371/journal.pone.0185514 (PMC5619773; doi:10.1371/journal.pone.0185514)

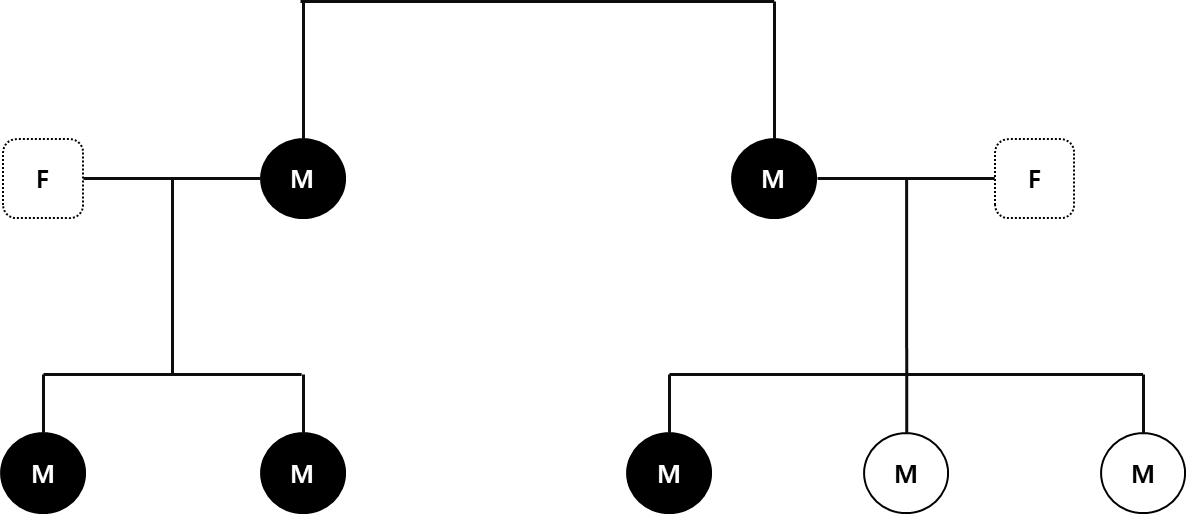

Supplement: S1 Fig — The person whose back-ground color is expressed in black is the patient, and the person expressed in white is the normal person. The person represented by the dotted line has no data. (TIF) [file pone.0185514.s001.tif]
