# Supplementary material for: Gene selection tool (GST): A R-based tool for genetic disorders based on the sliding-window proportion test using whole-exome sequencing data
Source: PLoS One. 2017 Sep 28;12(9):e0185514. doi: 10.1371/journal.pone.0185514 (PMC5619773; doi:10.1371/journal.pone.0185514)
Supplement: S1 File — It is a R file that contains R code, sample data and user guide. (ZIP) [file pone.0185514.s002.zip › GST User guide.pdf]

GST(Gene Selection Tool)

Description

GST() is a method to find the causative genes of specific diseases using WES data.

Identify the causative genes and show the location of the genes in figures and tables.

Usage

GST(x, state=NULL, parents=T, Trimmed.score=F, TOP=0)

Require Package

ggplot2
ggrepel
IRanges
GenomicRanges
Homo.sapiens
VariantAnnotation
TxDb.Hsapiens.UCSC.hg19.knownGene
Mygene
rfPred
curl

Arguments

x

Data from SNV of patients and normal people combined by chromosome and locus. If there is a parent's data, you must structure the data in chromosome, locus, reference, parental, and child data order.
In the column name, chromosome, locus, and reference are created as follows.
colnames(data)=c(Chr, Locus, Ref., father, mother,…)
Here, colnames “Chr, Locus, Ref.” are mandatory. The names of family members do not matter.

state

The patient and the normal person are represented by 1 and 0 respectively in the order of data x. (necessary)

parents

It is expressed as **parents=T** if there is parents data in data x, and **parents=F** if not.(necessary)

Trimmed.score

It is an option to trime the remaining scores except the selected genes for visual convenience when drawing the selected genes in pictures. To use the option, use Trimmed.score=T. (default : Trimmed.score=F)

TOP

It is an option used to select the top few of the selected genes. (Constant)

Details

The GST is designed to look for genes that cause specific families or specific diseases using SNV data. For families, specify parents = T when all parents have data, and parents = F if they are not. In the SNV data, list patients and normal individuals in order 1 and 0, respectively.

Although the window size is not fixed, we recommend the window size selection method of this paper. Probability test as a basic analysis method. Therefore, you can set the value of the probe option to determine the SNV pattern within the window size. Generally, it is based on 0.5, but for a more rigorous test, you can set a value less than 0.5.

The table shows all the genes selected by the test, but in the figure, only the top few gene can be seen using the TOP option. In addition, Trimmed.score = T can be used to make the selected gene easier to see.

(Each figures and tables are saved in “.png” and “.txt” files)

Examples

```
data=read.table('sampledata',header=T)

##################################
######### data example ################

Chr Locus Ref. P D1 D2 MS MSD1 MSD2 MSD3
chr1 13273 G C/C G/C C/C C/C G/C G/C G/G
chr1 13418 G G/A G/A G/G G/A G/A G/G G/A
chr1 13504 G G/G G/G G/G G/A G/A G/G G/A
chr1 14653 C C/C C/T C/T C/T C/T C/T C/T
chr1 14677 G G/G G/G G/G G/A G/A G/A G/A
chr1 14907 A A/A A/G A/G A/G A/A A/A A/G

#################################
############# Basic ###############

source('GST Code.R')
GST(data, state=c(1,1,1,1,1,0,0),parents=F)


############# Options ###############

GST(data, state=c(1,1,1,1,1,0,0),parents=F,TOP=10)
                                                    # Only TOP10 genes will appear.
GST(data, state=c(1,1,1,1,1,0,0),parents=F, Trimmed.score=T)
                                                    # Trimmed score plot
```

Session Information

R version 3.3.3 (2017-03-06)
Platform: x86_64-w64-mingw32/x64 (64-bit)
Running under: Windows 7 x64 (build 7601) Service Pack 1

locale:
LC_COLLATE=Korean_Korea.949
LC_CTYPE=Korean_Korea.949
LC_MONETARY=Korean_Korea.949 LC_NUMERIC=C
LC_TIME=Korean_Korea.949

attached base packages:
stats4, parallel, stats, graphics ,grDevices, utils, datasets, methods, base
